# Supplementary material for: Systematic genetics and single‐cell imaging reveal widespread morphological pleiotropy and cell‐to‐cell variability
Source: Mol Syst Biol. 2020 Feb 17;16(2):e9243. doi: 10.15252/msb.20199243 (PMC7025093; doi:10.15252/msb.20199243)
Supplement: Supplementary file 1 — Expanded View Figures PDF [file MSB-16-e9243-s001.pdf]

Expanded View Figures

Figure EV1. Assessing the feature space. Related to Fig 1, Table EV1.

A Hierarchical clustering of feature vectors composed of the average CellProfiler feature values across all single cells labelled for each phenotype. Average linkage and the Euclidian distance metric were used.

B High-dimensional feature space for single cells (colour-coded by phenotype) from the training sets visualized with 2D t-SNE. Numbers follow the phenotype order listed in Fig 1C.

© 2020 The Authors

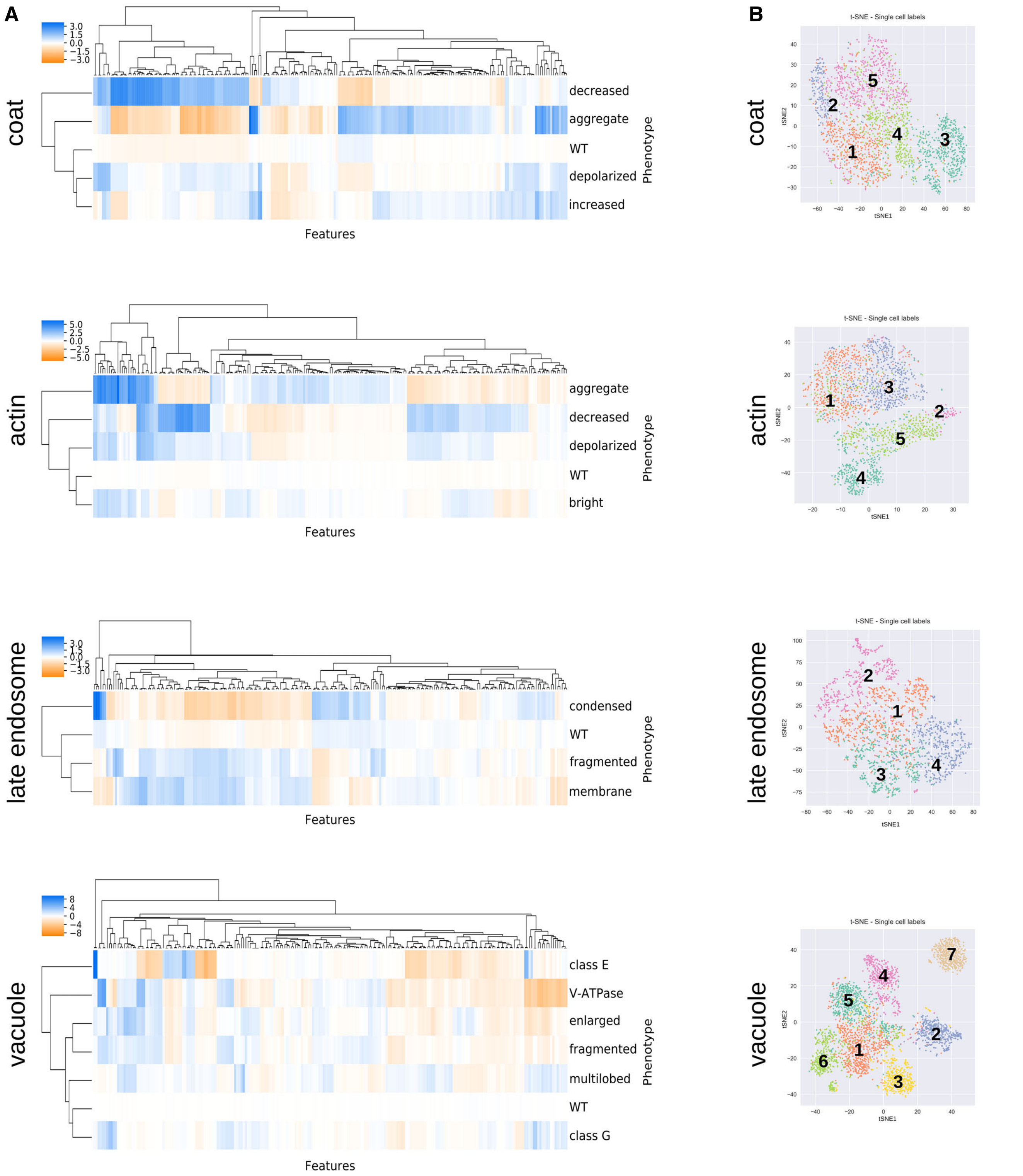
A
B
coat
decreased
aggregate
WT
depolarized
increased
Phenotype
Features
actin
aggregate
decreased
depolarized
WT
bright
late endosome
condensed
WT
fragmented
membrane
vacuole
class E
V-ATPase
enlarged
fragmented
multilobed
WT
class G
t-SNE - Single cell labels
tSNE1
tSNE2

Figure EV1.

Figure EV2. Factors affecting classification accuracy and penetrance. Related to Fig 1, Table EV1.

A Confusion matrices illustrating the classification accuracies of the 2NN classifiers for all phenotypes. Numbers in the matrix reflect the mean accuracy of both genome-wide and secondary screens. * denotes phenotypes where the difference in accuracy between the genome-wide and secondary screens was > 0.10. Numbers in brackets indicate the total number of labelled cells in the two filtered training sets for each phenotype. The classifiers for the two "aggregate" phenotypes (denoted[1,2]) were trained using < 100 labelled cells in one or both of the screen types. The intensity of the blue colour in each block of the matrix indicates the fraction of cells classified from each class predicted to be in a given class (scale bar to the right). Classification accuracy for each class is indicated by the number in each block.

B Scatter plot showing the 2NN classifier accuracy and number of labelled cells for each training set separately ($N = 42$), where each dot represents one phenotype class. No outline: training set for genome-wide screen. Black outline: training set for secondary screen.

C Comparison of the manually assigned and computationally derived penetrance of positive control strains (see Table EV1 for list of strains). Each dot represents one positive control from either the genome-wide (GW) screens (light blue dots) or secondary screens (dark blue dots), and grey dots are wild-type controls. LE = late endosome.

D Analysis of penetrance in biological replicates. The bar graph shows the fraction of biological replicates grouped according to their difference in penetrance ($N = 15{,}398$ replicate pairs). Less than 10% of replicates have a penetrance difference > 30 (grey bars), with an average penetrance difference of 11.2. Inset pie chart shows a breakdown of the underlying cause of large penetrance differences.

E Bootstrapping on wild-type cell populations to determine the number of cells sufficient to obtain a confident penetrance calculation. The shaded area indicates the range of the minimum sample size across the four screened markers (defined as the sample size where the relative standard deviation falls below 0.2). Data are presented as the mean penetrance across 100 independent samplings for each sample size (blue line) ± SD (error bars).

F Penetrance frequency distribution of wild-type replicates for each of the four markers extracted from genome-wide screening data. The shaded area indicates the mean (vertical dashed lines) $\pm 0.2 \times$ mean. Colours represent the different endocytosis markers as shown in the legend.

G Evaluation of possible batch effects in the penetrance analysis. Representations of two screened plates illustrating cell count (orange) and computationally derived penetrance (blue) in each well are shown. Empty wells are coloured grey. A darker shade of orange or blue indicates increased cell number or penetrance as shown on the key below the plate representations. Even though uneven growth conditions can lead to plate-layout effects, such as gradients (top plate) or more favourable edge conditions (bottom plate), the cell density differences due to experimental artefacts do not significantly affect penetrance analysis.

 © 2020 The Authors

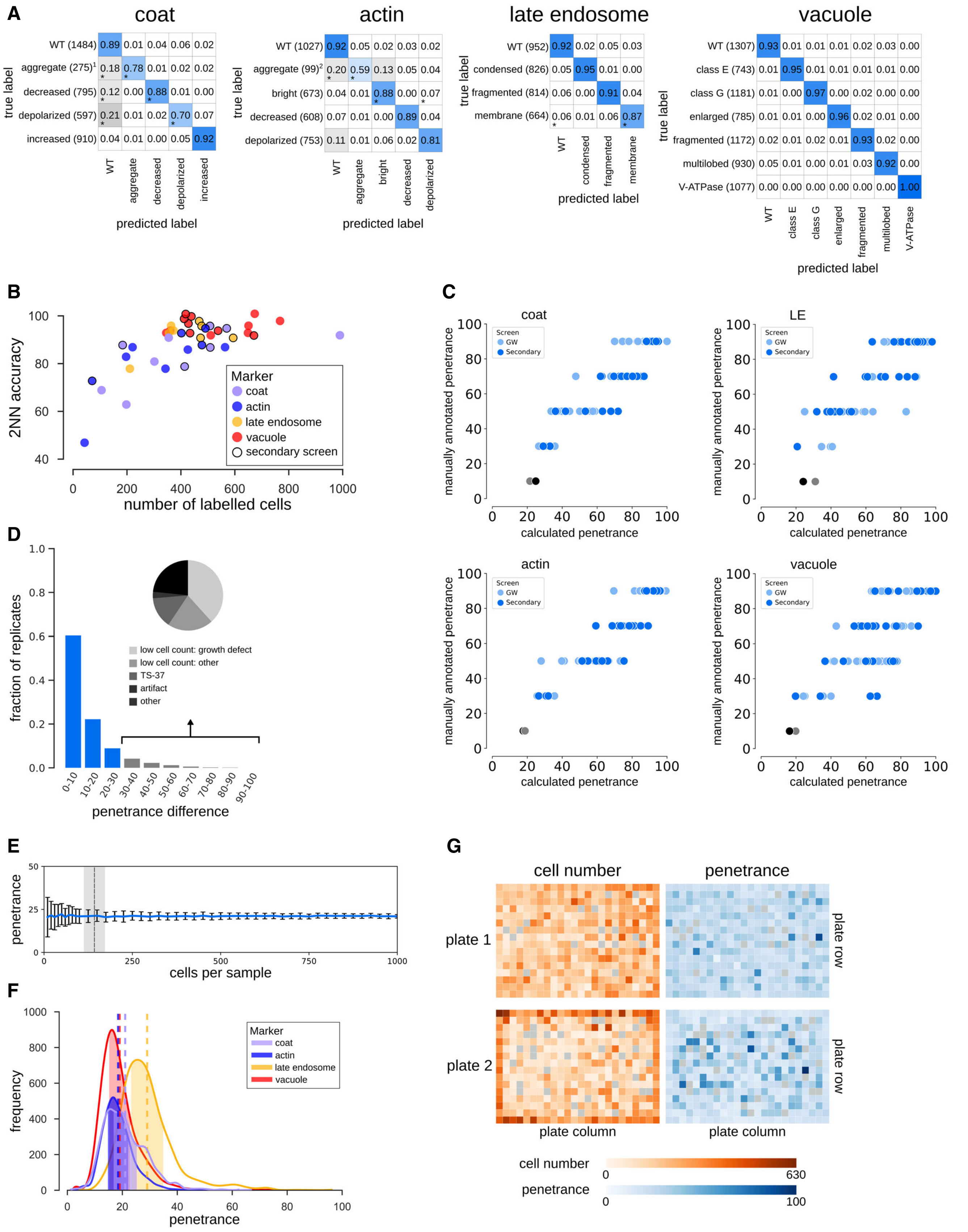
A
coat
WT (1484) 0.89 0.01 0.04 0.06 0.02
aggregate (275)1 0.18 0.78 0.01 0.02 0.02
decreased (795) 0.12 0.00 0.88 0.01 0.00
depolarized (597) 0.21 0.01 0.02 0.70 0.07
increased (910) 0.04 0.01 0.00 0.05 0.92
WT aggregate decreased depolarized increased
true label
predicted label
actin
WT (1027) 0.92 0.05 0.02 0.03 0.02
aggregate (99)2 0.20 0.59 0.13 0.05 0.04
bright (673) 0.04 0.01 0.88 0.00 0.07
decreased (608) 0.07 0.01 0.00 0.89 0.04
depolarized (753) 0.11 0.01 0.06 0.02 0.81
WT aggregate bright decreased depolarized
late endosome
WT (952) 0.92 0.02 0.05 0.03
condensed (826) 0.05 0.95 0.01 0.00
fragmented (814) 0.06 0.00 0.91 0.04
membrane (664) 0.06 0.01 0.06 0.87
WT condensed fragmented membrane
vacuole
WT (1307) 0.93 0.01 0.01 0.01 0.02 0.03 0.00
class E (743) 0.01 0.95 0.01 0.01 0.01 0.01 0.00
class G (1181) 0.01 0.00 0.97 0.00 0.02 0.00 0.00
enlarged (785) 0.01 0.01 0.00 0.96 0.02 0.01 0.00
fragmented (1172) 0.02 0.01 0.02 0.01 0.93 0.02 0.00
multilobed (930) 0.05 0.01 0.00 0.01 0.03 0.92 0.00
V-ATPase (1077) 0.00 0.00 0.00 0.00 0.00 0.00 1.00
WT class E class G enlarged fragmented multilobed V-ATPase
B
2NN accuracy
number of labelled cells
Marker
coat
actin
late endosome
vacuole
secondary screen
C
coat
LE
actin
vacuole
Screen
GW
Secondary
manually annotated penetrance
calculated penetrance
D
fraction of replicates
penetrance difference
low cell count: growth defect
low cell count: other
TS-37
artifact
other
E
penetrance
cells per sample
F
frequency
penetrance
Marker
coat
actin
late endosome
vacuole
G
cell number
penetrance
plate 1
plate 2
plate row
plate column
cell number 0 630
penetrance 0 100

Figure EV2.

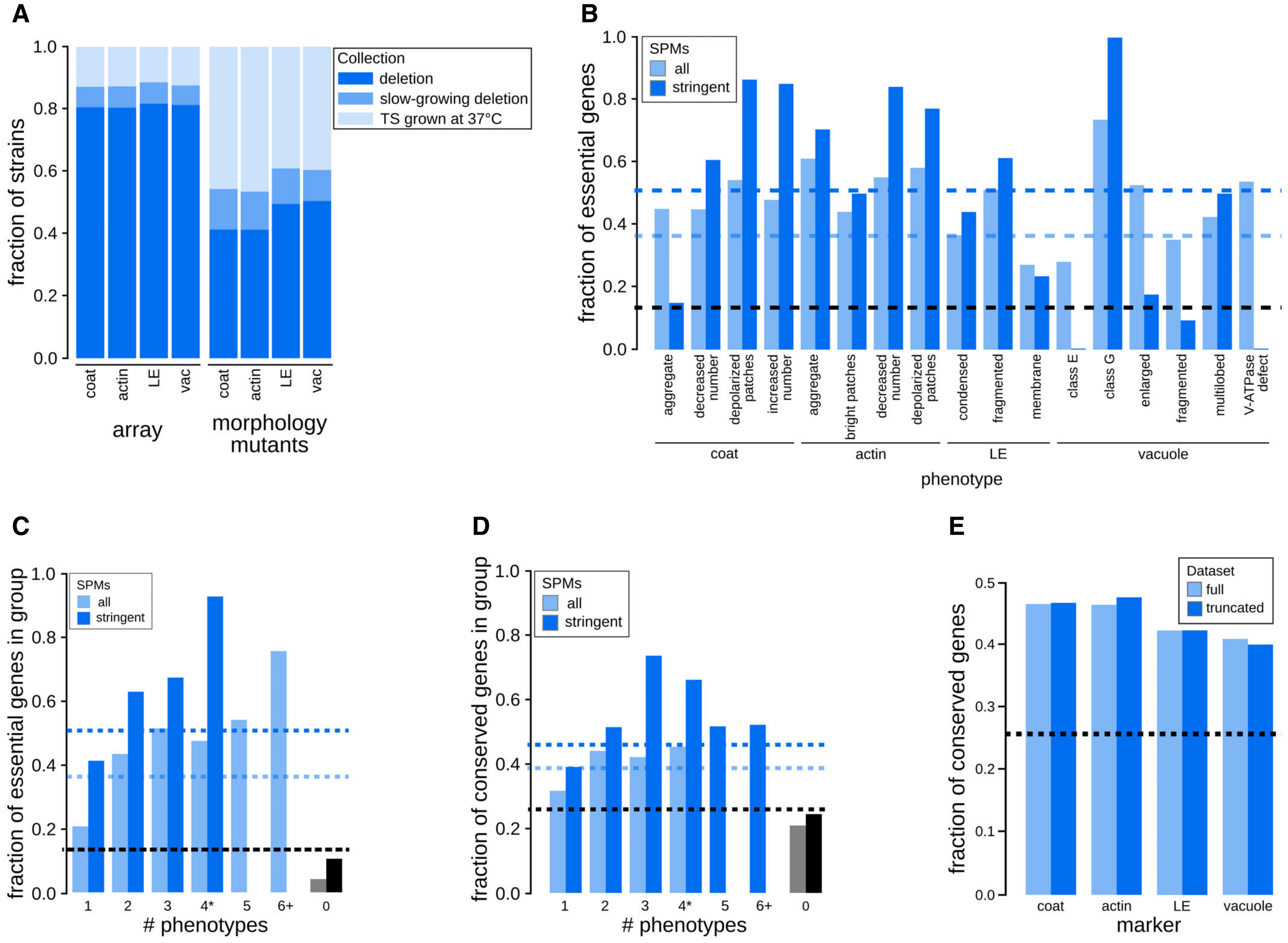

Figure EV3. Emerging properties of mutant phenotypes. Related to Fig 2, Table EV2.

A Comparison of the fraction of mutants screened and the fraction identified as morphology mutants in each strain collection. Stacked bar graphs show the fractions of strains in the screened array (array) and in the set of morphology mutants grouped based on the mutant strain collection for each individual marker (deletion mutant collection—dark blue; slow-growing non-essential gene-deletion collection—medium blue; collection of strains with TS mutations in essential genes—light blue). LE: late endosome; vac: vacuole.

B Relationship between specific phenotype mutants (SPMs) and essential genes. Bar graph showing the fraction of essential genes in sets of SPMs (light blue) and stringent SPMs (dark blue) for each individual phenotype. The black dashed line represents the fraction of essential genes in the screened mutant array. Blue dashed lines indicate the fraction of essential genes for all SPMs (light blue) and stringent SPMs (dark blue). LE: late endosome.

C Bar graph illustrating the relationship between essential genes and morphological pleiotropy. Bar graph showing the fraction of essential genes in specific phenotype mutants (SPMs; light blue) and stringent SPMs (dark blue) grouped by the number of phenotypes they affect. Blue dashed lines indicate the fraction of essential genes for all SPMs (light blue) and stringent SPMs (dark blue). The black dashed line represents the fraction of essential genes in the screened mutant array.

D Relationship between conserved genes and morphological phenotypes. Bar graph showing the fraction of conserved genes in specific phenotype mutants (SPMs; light blue) and stringent SPMs (dark blue) grouped by the number of phenotypes they affect. The black dashed line represents the fraction of conserved genes in the screened mutant array. Blue dashed lines indicate the fraction of conserved genes for all SPMs (light blue) and stringent SPMs (dark blue).

E Bar graph showing the fraction of conserved genes in our morphology mutant sets for each of the markers for the full dataset, and a truncated dataset with excluded genes annotated to GO Slim biological process terms associated with endocytosis and the endomembrane system. Black dashed line denotes the fraction of conserved genes in the screened mutant array. LE: late endosome.

 © 2020 The Authors

Figure EV4. Properties of specific mutant phenotypes. Related to Fig 3, Tables EV3 and EV6.

A Time-course analysis of vacuolar class G phenotype formation. Wild-type and *sec18-1* strains expressing Vph1-EGFP were first imaged at room temperature (RT), the temperature was then shifted to 37°C, and images were acquired at the indicated time points (in hours after shift). Signal intensity of the magnified insets (in solid boxes within the micrographs) was adjusted to optimize phenotype visualization. Scale bar: 10 μm.

B Gene feature enrichment analysis of the morphology mutants for each endocytic marker. Significance was determined using one-sided Mann–Whitney *U*-tests for numeric features, and one-sided Fisher's exact tests for binary features. For numeric features, dots represent median *z*-score normalized values. For binary features (below the solid black line), dots represent fold enrichment. Gene features derived from our genome-wide screens are indicated with "GW screen data" (shown above the black dotted line). CV: coefficient of variation. GI: genetic interaction. RV: relative variability. LE: late endosome; vac: vacuole.

C Heatmap of pairwise Pearson correlations between the 17 mutant phenotypes. A more intense blue colour indicates a higher PCC (scale bar at the top left). Unsupervised hierarchical clustering was performed using the correlation metric and average linkage.

D Horizontal box plot showing the distribution of endocytic internalization defect (invertase score as assessed in Burston *et al*, 2009) for non-essential specific phenotype mutants (SPMs). Several phenotypes show a significant difference between SPMs with a high specific phenotype fraction (dark blue circle) compared to those with a lower specific phenotype fraction (light blue circle). *, ** denote phenotypes with a significant difference between the two groups ($P < 0.05$ or < 0.01; significance was calculated using Kolmogorov–Smirnov tests). Black triangle: mean; central black lines: median; black dashed line: mean of phenotypically wild-type mutants. Numbers in the right-most column indicate the number of genes included in the analysis. Whiskers extend to the 5th and 95th percentile. LE: late endosome.

© 2020 The Authors

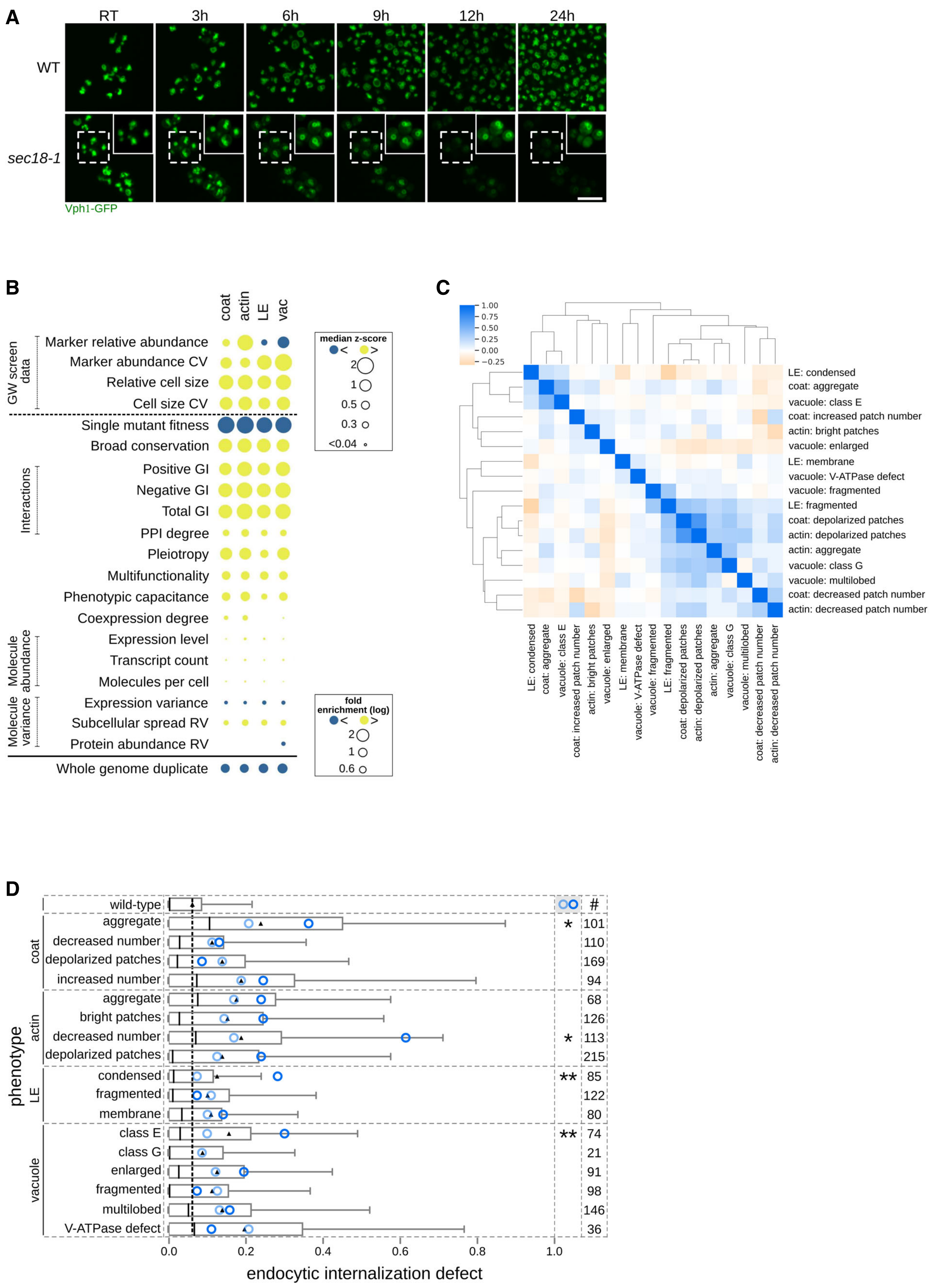

Figure EV4.

© 2020 The Authors

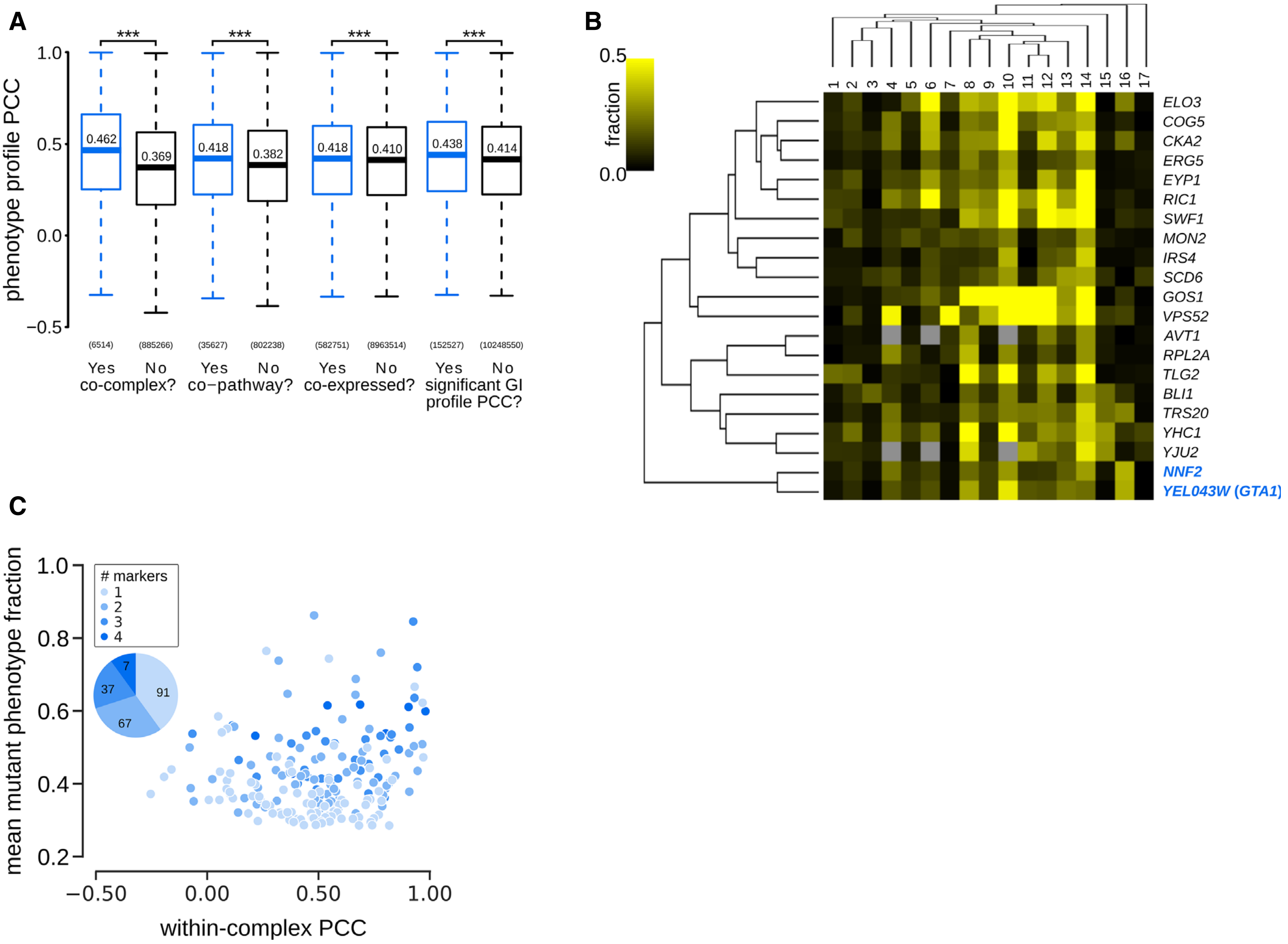

Figure EV5. Relationship between phenotype profiles and functionally related gene pairs. Related to Fig 4, Table EV7.

A Phenotype profile similarity of functionally related pairs of genes. Box plot indicates the distribution of Pearson correlation coefficients (PCCs) between pairs of specific phenotype profiles for genes encoding members of the same or different protein complex (co-complex); proteins in the same or different pathway (co-pathway); genes that are co-expressed or not (co-expressed), and gene pairs that have a significant GI profile similarity or not (significant GI profile PCC). The box represents IQR (interquartile range). Whiskers are Q1-1.5*IQR and Q3+1.5*IQR. Central lines represent the median. The number of pairs evaluated in each set is shown on the *x*-axis. Significance was determined using one-sided Mann–Whitney *U*-tests. ****P* < 0.001.

B Phenotype profile cluster containing *NNF2* and *YER043W* (*GTA1*) (highlighted in blue). Phenotypes 1–17: [1] coat: increased patch number; [2] coat: aggregate; [3] vacuole: class E; [4] late endosome: condensed; [5] actin: bright patches; [6] late endosome: membrane; [7] actin: aggregate; [8] coat: decreased patch number; [9] actin: decreased patch number; [10] late endosome: fragmented; [11] coat: depolarized patches; [12] actin: depolarized patches; [13] vacuole: multilobed; [14] vacuole: fragmented; [15] vacuole: enlarged; [16] vacuole: class G; [17] vacuole: V-ATPase defect. Fraction: specific phenotype fraction.

C Relationship between protein complexes and morphological phenotype profile correlations. Scatter plot showing mean mutant phenotype fraction (*y*-axis) and mean within-complex phenotype profile PCCs (Pearson correlation coefficient; *x*-axis) for individual protein complexes (*n* = 202). The inset pie chart shows the proportion (and number) of protein complexes that affect 1, 2, 3 or all 4 markers. Mean penetrance was calculated only from affected markers. Complexes are colour-coded based on the number of markers they affect.

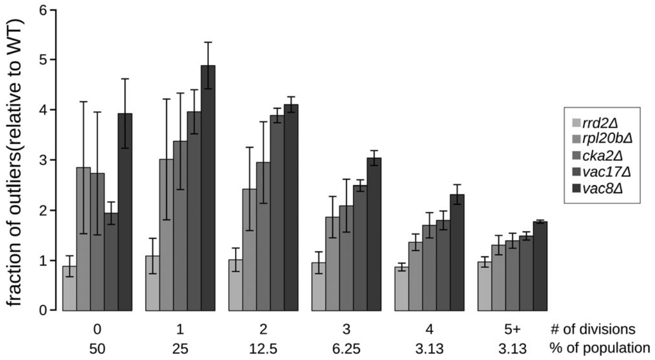

Figure EV6. Penetrance as a function of replicative age. Related to Fig 6, Table EV8.

Bar graph showing the fraction of outliers relative to wild type in populations of increasing replicative age (# of divisions) for 5 mutant strains (*rrd2*Δ, *rpl20b*Δ, *cka2*Δ, *vac8*Δ and *vac17*Δ). Data are presented as mean of three biological replicates ± SD.

 © 2020 The Authors
